# Healthily Nourished but Depleted? Is It Possible to Improve the Health of Shift Workers through Lifestyle Interventions?

**DOI:** 10.3390/bs14060454

**Published:** 2024-05-28

**Authors:** Christine Binder-Mendl, Cem Ekmekcioglu, Wolfgang Marktl, Thorsten Schwerte

**Affiliations:** 1 Institut für Zoologie, Universität Innsbruck, Hochfügenerstraße 61, A-6263 Fügen, Austria; 2 Department of Environmental Health, Center for Public Health, Medical University of Vienna, Kinderspitalgasse 15, A-1090 Vienna, Austria; cem.ekmekcioglu@meduniwien.ac.at; 3 Wiener Internationale Akademie für Ganzheitsmedizin, Otto Wagner Spital Sanatoriumstrasse 2/Gebäude G, A-1140 Vienna, Austria; marktl@gamed.or.at; 4 Institut für Zoologie, Universität Innsbruck, Technikerstraße 25, A-6020 Innsbruck, Austria; thorsten.schwerte@uibk.ac.at

**Keywords:** chronobiology, rotating shift work, questionnaire SF-36, mental health, nutritional counselling, physical activity

## Abstract

The relationship between diet and health is well-researched, and there is also information regarding the effects of diet on mental health. This study aimed to investigate whether motivation to optimize lifestyles without regulations or restrictions could improve the health of rotating shift workers. In this pilot study, 18 male shift workers were randomly divided into two groups. All participants completed the Short Form Health Survey-36 questionnaire (SF-36) before the start and at the end of the study. Group I (n = 9, mean age 42 ± 6.6 y) received dietary and lifestyle information every other month for one year, and the other, Group II (n = 9 mean age 36 ± 7.3 y), one year later. All participants were motivated to follow the trained dietary recommendations and to engage in physical activity. Almost all scores had improved. Surprisingly, physical performance scores worsened, which was not expected. The impairment in mental health due to the change in ownership of the company could have been better explained. Nutritional advice over a longer period and the motivation to integrate more exercise into everyday life can potentially improve the health of rotating shift workers.

## 1. Introduction

Being asked to work at peak performance at any time of the day means additional stress for about 18% (USA) [1] to 21% (Europe) [2] of the working population [3]. While workplaces with flexible working hours are a good way of achieving peak performance without sacrificing the quality of life, working alternating shifts with day and night changes is a challenge, especially when ‘owls’, means people who prefer to get up late in the morning and go to bed late in the evening, are expected to prove their performance early, before sunrise [4]. Working at night, when all the body’s signals are tuned for rest, recovery, and sleep, not only leads to stress but also carries the risk of fatigue with all its consequences [5,6].

This not only entails the risk of creating sources of error but can also affect the health of shift workers [7,8]. It is well-known that rotating shift work is associated with a higher risk of illness, not only in terms of physical health [9] but also considering mental health [3,10,11,12,13]. Since life-sustaining jobs cannot be performed without night working, increased attention should be paid to workers in these occupations. Previous studies have shown that the state of health becomes increasingly worse the longer the shift work is performed [14]. Therefore, precautions should be taken at an early stage to achieve a health-promoting effect.

The social environment at work has a significant impact and contributes to the health of shift workers [15]. However, the most effective and cost-efficient health promotion methods are changes to one’s lifestyle [16]. The foundation for healthy aging is laid by a healthy lifestyle and nutrition plays an important role in lifestyle changes [17]. A healthy diet cannot prolong life, but it can help prevent illness and enable healthy aging. It is the combination of different, mainly plant-based foods that has a positive effect on health and it depends on how much we eat of them and when [18]. The negative effect of food intake during the night is well-described [19,20,21,22] but it is also a sensitive topic, because eating often means more than just energy intake [23]. Meals are sometimes the only opportunity for a family to come together and thus signify community, but they are also part of culture and social status [24,25]. To achieve a change in eating habits, this fact must also be taken into account. Shift workers, in particular, lack this important communicative exchange because, due to their changing work schedules, they cannot always attend family meals. For these reasons, nutritional counselling must be carried out very carefully and sensitively if a change is to take place.

Weyh [26] reported the health-promoting effect of physical exercise, not only considering body weight but on overall health as well as sleep quality [27]. Although alternate shifts have less impact on sleep quality than permanent night shifts [5], they do influence sleep duration and recovery during sleep. Reduced sleep quality and sleep duration can lead to psychological disorders such as burnout, depression, or suicidal ideation [28], which not only impair the quality of life of shift workers but can also have a lasting negative impact on their health.

Since shift work has become an indispensable part of our working world, people who work both day and night deserve more attention and care than the rest of the working population, considering more than just the healthcare sector. There is a consensus on the physical effects, such as the deterioration in sleep quality and even insomnia, the tendency to obesity and diabetes type 2, and the susceptibility to cardiovascular disease among shift workers, but opinions differ on the psychological consequences. While Berthelsen et al. [10] did not see any differences in the psychological burden of shift workers, Bara and Arber [14] found that men who worked night shifts suffered from mental health problems more frequently and more severely than women.

This fact was the motivation for this study, which involved only male shift workers, with the aim to try to improve their health status by providing nutritional information and recommendations for regular exercise without limiting their quality of life [29]. It is important to emphasize that this study did not prescribe any special or restrictive diets. The subjects were simply informed about healthy eating as recommended by nutrition societies. Nutritional information without rules and restrictions, as well as the integration of physical activity and muscle-strengthening training [30,31,32] into the daily routine, was intended to improve the health of the subjects in the long term. One aim of this present study was to steer lifestyles in a healthier direction; another target was to find out whether the information about healthy eating and incorporating more exercise into daily routines would persist even when no further advice was given.

## 2. Methods

### 2.1. Participants

**Figure 1:**
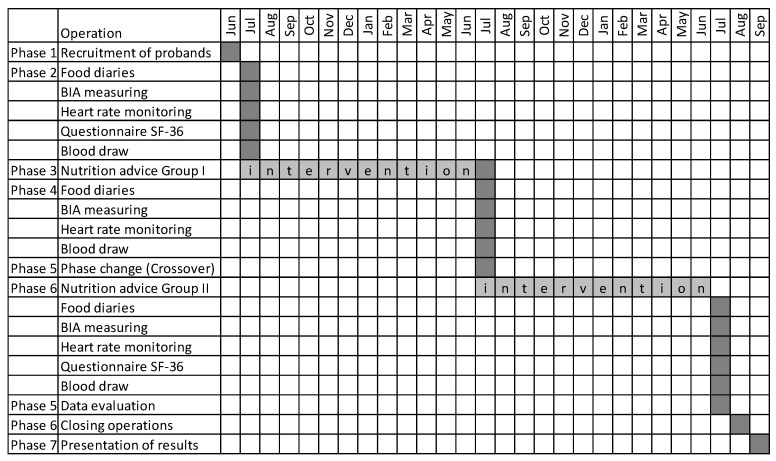
The test plan shows the schedule, scope and procedure of the study (2019–2021). The grey box indicates the time period in which the action was performed.

After the approval by the Ethic Commission, the study started with the recruitment of participants over four weeks. To recruit the participants (2019), all department heads of the company were informed in an e-mail that a study on workplace health promotion was to be conducted. All persons who expressed an interest in the study were included. Subsequently, an informative meeting was held in the company’s meeting room by the leader of the study to inform all present employees about the implementation and procedure of this study. The following month, a meeting was arranged with the subjects in the office of the company’s physician. During this meeting, the subjects signed the consent form, they were given a nutrition diary, and an appointment was made. We could assume an identical level of education because they were workers from the company with identical education and qualifications. The twenty male participants were rotating shift workers from an energy-generating company. In this study, only eighteen questionnaires could be evaluated because one subject had difficulties with the German language and another participant quit working in shifts. The subjects (n = 18 mean age 37.4 ± 7.5 y) were divided into two groups, based on the urn model. After they had been briefed about the proceedings, the participants signed the informed consent. While Group I (n = 9) received nutritional counselling every two months in the following year, Group II (n = 9) was told that their intervention would start one year later. The procedure aimed to investigate the sustainability of a lifestyle intervention. The instructed subjects were encouraged to use the time between the consultations to integrate the given advice in their daily routine. One year after the study started, it was complicated by the fact that the owner of the company changed, which contributed to uncertainty in the subjects. The study procedure is described in the test plan (see Figure 1 below).

### 2.2. Lifestyle Intervention

#### 2.2.1. Nutrition

The provided nutritional information was based on the recommendations of a dietitian and nutritionist (more detailed information can be found in [29]). 

The subjects in the present study were allowed to freely choose the type and amount of food they consumed during a night shift. They recorded their diet in a one-week diary, once before the intervention and once at the end of the study, after the intervention. During the dietary counselling, they were advised to eat three meals a day: breakfast, lunch, and dinner, regardless of which shift the subjects worked. Regular meals prevent food cravings [33]. During cravings, larger quantities and more fatty and sugary foods are consumed. Between these main meals, it was recommended not to eat anything, especially at night, as it is known from previous studies [34] that shift workers are more likely to eat high-sugar and high-fat snacks during the night shift. If it could not be avoided, they were told to eat bite-sized vegetables, brought from home in a well-sealed bowl or vegetable soup if a hot meal was preferred. The subjects were also instructed to stick to fixed meal times for breakfast, lunch, and dinner, regardless of whether they had early, late, or night shifts. Half of the main meal was advised to consist of vegetables and salads, a quarter was to be a carbohydrate-rich side dish, and the rest was advised to be meat, fish, or eggs. Since foods derived from animals should not be eaten every day, they are replaced by vegetables, legumes, or salad [17]. Regular meals are intended to prevent food cravings and promote healthy food choices. The subjects were advised to eat regionally, seasonally, and colorfully to ensure essential micronutrient intake. If something sweet was desired, it was to be enjoyed as a dessert in the form of fruit or a fruit salad. Expensive, individually wrapped small sweets or miniature-sized snacks should replace bulk packs as well as chocolate bars, automatically reducing calorie, fat, and sugar intake without renouncement. 

The intervention, based on this food log, enabled individual personal nutritional counselling and detection of dietary errors and food intolerances. Each subject was informed in an individual interview about healthy eating how foods are absorbed and processed in the body, which foods should be consumed daily to meet needs, and which should be consumed less frequently as well as the rationale for doing so. In a practical exercise, participants estimated how much fat or sugar is contained in commonly used foods and learned what to look for when shopping. Their senses were trained as they blindly guessed foods, identified dried fruits by taste, and guessed herbs and spices by scent.

The analysis of the dietary protocol was calculated with the dietary calculation program Aconsoft PIU Printex GmbH, Vienna, Austria; Acon BKVBLS 2014; database BLS II.2 and BLS II.3, once at the beginning and once at the end of the study. Based on this procedure, changes in the intake of macro- and micronutrients as well as dietary fiber and alcohol consumption could be determined [29].

To assess the health of the subjects objectively, nutritional blood parameters were measured at baseline and at the end of the study. Although the blood test is only a snapshot of nutritional status, it is often used to determine nutrition-related health parameters [35]. To determine the actual nutritional status, a biopsy would need to be taken, which is not performed on healthy individuals. Since this pilot study is only a comparison of the initial situation with the end of the study, the blood values were used as a basis. The results have already been published [29]. The majority of parameters improved, in some cases even significantly.

#### 2.2.2. Physical Activity

A healthy lifestyle includes not only paying attention to nutrition but also taking care of the body. That is why advice for a healthy lifestyle must always take physical activity into account. Strength training prevents age-related muscle loss and increases the basic metabolic rate. Endurance training helps to maintain muscle mass. Participants were not only asked to change their eating habits but also to incorporate more exercise into their daily routine. While some subjects attended a gym regularly, others had to be encouraged to ride their bikes or go for a walk. They were shown simple exercises and the proper use of home resistance bands, which were provided as needed.

### 2.3. Statistics

The Statistical Package for the Social Sciences IBM SPSS Statistics version 24 was used for statistical analysis. The Mann–Whitney U test for independent samples was employed to compare the results of Groups I and II. The Wilcoxon test for related samples was used for comparison of the results within the groups before and after intervention. The statistical significance was set to *p*-value ≤ 0.05. 

### 2.4. Health Status Questionnaire

The Short Form (SF-36) Health Survey is a questionnaire in which the participants define questions about their state of health. In this study, the SF-36 in the second supplemented and revised edition by Matthias Morfeld, Inge Kirchberger, and Monika Bullinger and published by Hogrefe Verlag Göttingen was used in the German language. This form of self-documentation was important to the authors to investigate whether steering lifestyle in a healthier direction can have a lasting effect without restricting the quality of life of the subjects. In this study, the questionnaire was mainly used to compare the state of health at the beginning and end of the study [36].

## 3. Results

Eighteen of the initial twenty questionnaires could be evaluated (90%). However, the results were not significant. One subject had language difficulties with the questionnaire. A vocal translation was made but this may have changed the subjective perception of this participant. Another subject left the company after one year and chose a job without working in shifts. For these reasons, the two questionnaires were excluded from evaluation.

Comparing the results of both groups combined at the beginning and the end of the study, an improvement of all results except for physical functioning can be seen (Table 1). 

Regarding the groups separately, the outcome was quite different. While in Group I, the physical and emotional role function increased to the maximum value until the end of the study, these scores decreased in Group II (Table 2). Furthermore, in Group I, vitality and mental well-being declined and physical functioning stayed the same, while in Group II, physical functioning and physical pain decreased too, which resulted in a lower standardized physical summation scale.

When comparing the groups at the start of the study, a completely different result would have been expected. While Group I started with six lower scores than Group II, it finished with an increase in physical and mental summation scale (Table 2). Group I was able to improve both physical and mental summation scales. 

Group II ended the study with seven deteriorated scores in comparison with Group I, although the average age in Group II was six years less than in Group I (Table 3). In Group II, the physical summation scale worsened by the end of the study.

## 4. Discussion

The authors are aware that the results of this pilot study can only be evaluated with caution because of the small number of subjects. Due to time and financial constraints, as well as the voluntary nature of participation in this study, it was not possible to recruit additional participants. Nevertheless, the present study was able to show that it is possible to push the attention of shift workers in a healthier direction by changing their diet [37] with a nutrition intervention program [38,39]. 

Given the participants’ good starting position, it was a challenge to achieve an improvement. Some participants joined the study because they wanted to improve their performance. They hoped that changing their eating habits would give them a chance to improve their ranking. Others were trying to maintain their weight after quitting smoking. Another reason for participating was to lose weight, if subjects were overweight. While some took it for granted to go for a walk or to work out at a gym regularly, others had to be encouraged. The need for regular strength training was only understandable to some when the meaning behind it was explained. Resistance bands were provided so that it could also be done at home.

Although the results did not reach statistical significance and the reasons for participation varied widely, improvements were achieved with minimal financial and human resources [40,41]. The consultations were adapted to the subjects’ shift schedules. They took place before or after shift work so that the participants did not have to make additional trips and invested as little time as necessary. Sometimes, counselling sessions were also conducted at the subjects’ homes if they did not have a babysitter available. The multiplier effect of the consultation on the family members was an advantage. On-site, products frequently used in the household could also be examined right away.

The change of ownership was an unpredictable event that may have been an additional stressor. The relationship between diet, stress, and physical and mental health is well-established [37] but not yet fully explored. Shift work is a stressor in itself. The subjects in this study were additionally stressed by the change in management at their company, which brought uncertainty about keeping their jobs. This anxiety was unfounded, however, as all subjects who wished to do so retained their jobs.

The influence of diet on health is well-researched [33,42]. Research about the role of nutrition in mental health was previously lacking [18]. However, research continues to emerge demonstrating the importance of eating habits on psychological health [18]. Nutrition is part of human culture. Therefore, people try to maintain their eating habits even when they leave their familiar environment. A change in eating habits can act as a stressor [43,44,45], as it would require people to change a significant part of their lives. For this reason, dietary advice must be given very carefully and sensitively, and it has been shown that intensive nutritional intervention [46] can have positive effects [18,47,48] if they are individualized. In this study, it is shown that individual nutritional advice, given a longer period, can improve the state of health [29,49].

It was interesting to note that the subjects found it much easier to change their eating habits than to incorporate endurance and strength training into their daily routine. In particular, the early shift from 06:00 h to 14:00 h was reported to be more stressful by all participants, not only by subjects who preferred to sleep longer in the morning. Berthelsen et al. [10] found no significant differences between the stress levels of different shift schedules. However, Ganesan et al. [6] found a correlation between sleep disturbances in rotating shift workers on early day shifts and those on night shifts, especially when these followed an evening shift. The reasons for this observation could include childcare, stressful morning routines, or a higher workload during those hours. Answering this question could be the topic of further study.

This early shift had a demotivating effect on their training units, although participants were told that physical activity can improve their sleep quality, as found in previous research [27]. Frequency [50] and the time of day when the training took place seemed to be less important than the sequence of the endurance and strength training [31]. 

While the connection between shift working people and overweight is debated [51], the impact of shift work on mental health is uniformly documented among experts [12,13]. Therefore, a negative psychological effect on the subjects after the change of leadership of the company would have been comprehensible. However, Group I in this study showed just a small deterioration in mental well-being and Group II worsened in terms of their emotional role function, but nevertheless, both groups improved the standardized mental summation scale of the short-form health survey until the end of the study.

The SF-36 is controversial and should not be used as the sole measure of health status. In this pilot study, it was only used to compare the baseline situation with the final result of the study [36]. Comparing the scores of the short-form health survey of both groups at the beginning and at the end of the study, only the score “physical functioning” had deteriorated, while all other scores improved. Bara and Arber [14] described the negative effects of night work on the mental health of men. Larry Culpepper [52] discovered that sleep deprivation and associated fatigue can lead to increased susceptibility to errors, which can have fatal consequences in some professions. These negative effects of shift work are not consistent with the results of the present study. None of the participants lost their jobs after the change in management, although everyone was afraid of it. Regardless of how long each participant worked at the company, everyone appreciated his work. One explanation for this could be that the rotating shift system is more friendly concerning work and family [53].

As expected, outcomes were better in Group I, where the intervention took place immediately after the start of the study, than in Group II, where the intervention took place one year after the start of the study, when the company had changed ownership. Contrary to expectations, the deteriorations were mainly in physical functions and less in mental functions. 

## 5. Limitations

The main limitation of the study is the number of subjects. As this was a voluntary study, it was not possible to increase the number of participants, although the study was presented to the entire workforce. 

Only the results of the food diaries during the night shift were taken into account. A comparison with the day shift would make the significance of the food intake more objective. Additionally, documenting all consumed food was perceived as stressful, so this was not done. 

The change in company management represents an additional limiting effect. The long-term intervention attempted to compensate for this but should be controlled by eliminating this effect. 

Attempts were made to fill the vending machines with at least one healthy snack or drink. As the owner of these vending machines wanted to secure sales, this form of intervention was not possible. With financial support from the management, it might have been successful. 

The company has a company canteen, which was only visited sporadically by the subjects. If the participants were offered a healthy menu at a lower price, they would perhaps eat more often in the canteen instead of snacking on something.

## 6. Conclusions

The present study shows that lifestyle modification can be achieved by providing personal and individual information about healthy foods without prohibitions, restrictions, or diets from a dietitian or nutritionist and by motivating daily exercise. Recurrent counselling at regular intervals could contribute to the health promotion of shift workers in a very cost-effective way.

## Figures and Tables

**Table 1:** The SF-36 scores of both groups combined at the start and the end of the study.

SF-36 Scores	Start of the Study	End of the Study	*p*-Value	
	(mean ± SD)	(mean ± SD)		
physical functioning	96.1 ± 5.3	95.6 ± 6.2	*p* = 0.925	↓
physical role function	94.4 ± 13.7	95.8 ± 17.7	*p* = 0.620	↑
physical pain	84.2 ± 18.0	88.1 ± 20.4	*p* = 0.461	↑
general health perception	76.8 ± 15.0	79.7 ± 13.7	*p* = 0.583	↑
vitality	66.0 ± 18.0	70.6 ± 16.4	*p* = 0.383	↑
social functioning	85.4 ± 25.5	94.4 ± 20.7	*p* = 0.301	↑
emotional role function	90.7 ± 22.3	94.4 ± 23.6	*p* = 0.620	↑
mental well-being	79.3 ± 17.5	81.1 ± 18.0	*p* = 0.904	↑
standardized physical summation scale	54.2 ± 4.6	54.7 ± 4.8	*p* = 0.678	↑
standardized mental summation scale	51.2 ± 10.7	53.6 ± 9.3	*p* = 0.718	↑

Numbers refer to the mean value (mean) and standard deviation (±SD) in a score from 0 to 100, while 0 represents the greatest possible restriction, and 100 means no limitation at all. ↑ values have improved; ↓ values have worsened. Differences were not significant.

**Table 2:** SF-36 scores of Group I at the start and the end of the study.

SF-36 Scores Group I	Start of Study	End of Study	*p*-Value	
physical functioning	95.0 ± 5.6	95.0 ± 7.5	*p* = 1.000	=
physical role function	88.9 ± 18.2	100.0 ± 0.0	*p* = 0.102	↑
physical pain	79.0 ± 20.7	91.1 ± 14.4	*p* = 0.068	↑
general health perception	78.7 ± 14.8	81.4 ± 10.1	*p* = 0.491	↑
vitality	68.9 ± 18.3	68.3 ± 16.4	*p* = 0.933	↓
social functioning	83.3 ± 30.0	98.6 ± 4.2	*p* = 0.102	↑
emotional role function	88.9 ± 23.6	100.0 ± 0.0	*p* = 0.180	↑
mental well-being	80.9 ± 19.1	79.1 ± 20.1	*p* = 0.395	↓
standardized physical summation scale	52.9 ± 4.4	55.6 ± 4.4	*p* = 0.441	↑
standardized mental summation scale	52.0 ± 11.4	53.8 ± 6.2	*p* = 0.859	↑

Numbers refer to the mean value (mean) and standard deviation (±SD) in a score from 0 to 100, while 0 represents the greatest possible restriction, and 100 means no limitation at all. ↑ values have improved; ↓ values have worsened; = values remained the same.

**Table 3:** SF-36 scores of Group II at the start and the end of the study.

SF-36 Scores Group II	Start of Study	End of Study	*p*-Value	
physical functioning	97.2 ± 5.1	96.1 ± 4.9	*p* = 0.317	↓
physical role function	100.0 ± 0.0	91.7 ± 25.0	*p* = 0.317	↓
physical pain	89.3 ± 14.2	85.1 ± 25.6	*p* = 0.336	↓
general health perception	74.9 ± 15.8	77.9 ± 16.9	*p* = 0.513	↑
vitality	63.1 ± 18.2	72.8 ± 17.2	*p* = 0.169	↑
social functioning	87.5 ± 21.7	90.3 ± 29.2	*p* = 0.564	↑
emotional role function	92.6 ± 22.2	88.9 ± 33.3	*p* = 0.317	↓
mental well-being	77.8 ± 16.9	83.1 ± 16.6	*p* = 0.395	↑
standardized physical summation scale	55.5 ± 4.7	53.9 ± 5.3	*p* = 0.594	↓
standardized mental summation scale	50.4 ± 10.7	53.4 ± 12.0	*p* = 0.314	↑

Numbers refer to the mean value (mean) and standard deviation (±SD) in a score from 0 to 100, while 0 represents the greatest possible restriction, and 100 means no limitation at all. ↑ values have improved; ↓ values have worsened.

## Data Availability

Raw data are available from the author named first.

## Abbreviations

SF-36—The Short Form Health Survey is a measurement instrument for the survey of the self-reported health-related quality of life of the participants. By answering the 36 questions, it describes the individual state of health and compares the course at the start and the end of the study.
